# RSC96 Schwann Cell Proliferation and Survival Induced by Dilong through PI3K/Akt Signaling Mediated by IGF-I

**DOI:** 10.1093/ecam/nep216

**Published:** 2011-06-08

**Authors:** Yung-Ming Chang, Wu-Hsien Kuo, Tung-Yuan Lai, Ying-Ting Shih, Fuu-Jen Tsai, Chang-Hai Tsai, Wen-Tong Shu, Ying-Yu Chen, Yueh-Sheng Chen, Wei-Wen Kuo, Chih-Yang Huang

**Affiliations:** ^1^Graduate Institute of Chinese Medical Science and Institute of Basic Medical Science, China Medical University, No 91, Hsueh-Shih Road, Taichung 404, Taiwan; ^2^School of Chinese Medicine, China Medical University, Taiwan; ^3^Division of Gastroenterology, Department of Internal Medicine, Armed-Force, Taichung General Hospital, Taiwan; ^4^School of Post-Baccalaureate Chinese Medicine, Taiwan; ^5^Department of Pediatrics, Medical Research and Medical Genetics, China Medical University, Taiwan; ^6^Department of Healthcare Administration, Asia University, Taiwan; ^7^Departments of Internal Medicine, Taiwan; ^8^Department of Orthopedics, Armed Force, Taichung General Hospital, Taiwan; ^9^Department of Biological Science and Technology, China Medical University, Taiwan; ^10^Graduate Institute of Basic Medical Science, China Medical University, Taiwan; ^11^Department of Health and Nutrition Biotechnology, Asia University, Taichung, Taiwan

## Abstract

Schwann cell proliferation is critical for the regeneration of injured nerves. Dilongs are widely used in Chinese herbal medicine to remove stasis and stimulate wound-healing functions. Exactly how this Chinese herbal medicine promotes tissue survival remains unclear. The aim of the present study was to investigate the molecular mechanisms by which Dilong promote neuron regeneration. Our results show that treatment with extract of Dilong induces the phosphorylation of the insulin-like growth factor-I (IGF-I)-mediated phosphatidylinositol 3-kinase/serine-threonine kinase (PI3K/Akt) pathway, and activates protein expression of cell nuclear antigen (PCNA) in a time-dependent manner. Cell cycle analysis showed that G_1_ transits into the S phase in 12–16 h, and S transits into the G_2_ phase 20 h after exposure to earthworm extract. Strong expression of cyclin D1, cyclin E and cyclin A occurs in a time-dependent manner. Small interfering RNA (siRNA)-mediated knockdown of PI3K significantly reduced PI3K protein expression levels, resulting in Bcl_2_ survival factor reduction and a marked blockage of G_1_ to S transition in proliferating cells. These results demonstrate that Dilong promotes the proliferation and survival of RSC96 cells via IGF-I signaling. The mechanism is mainly dependent on the PI3K protein.

## 1. Introduction

Nerve regeneration is a complex physiological response that takes place after injury. In mammals, central neurons without myelin sheaths are very difficult to regenerate. In contrast, the axons of neurons in the peripheral nervous system are surrounded by myelin sheaths and are, therefore, easier to regenerate [1]. Immature Schwann cells differentiate into a myelinating phenotype, laying down a myelin sheath around the axon of neurons in the peripheral nervous system, as well as proliferate and migrate into the injured nerve area to support axonal re-growth [2]. Schwann cell proliferation is crucial for successful nerve regeneration [3]. However, little is known about the signaling mechanisms that regulate Schwann cell proliferation.

Insulin-like growth factor-I (IGF-I) is a polypeptide hormone synthesized by proliferating Schwann cells [4]. It is secreted in response to growth hormone to stimulate tissue growth [5]. The IGF-I level elevation increases sympathetic neuron proliferation *in vivo* [6]. In addition, IGF-I stimulates the growth and differentiation of fetal neurons [7] and increases neurite sprouting and outgrowth *in vitro* [8, 9]. IGF-I has been shown to function as a progression factor in the cell cycle [10], promoting G_1_/S cell cycle progression via the phosphatidylinositol 3-kinase/serine-threonine kinase (PI3K/Akt) pathway, resulting in DNA synthesis and cell proliferation [11]. Interestingly, IGF-I not only stimulates proliferation but also promotes survival in a number of cell types [12]. Moreover, IGF-I acts as a therapeutic target for the treatment of peripheral nerve injury and motor neuron diseases [13]. This hormone protects neurons in the peripheral nervous system from apoptosis by activating the PI3K/Akt pathway, which in turn phosphorylates Bad and activates Bcl_2_, an anti-apoptotic protein that interferes with the activation of caspases [14–16]. *In vivo*, the signal cascade for early upregulation of IGF-I was shown to promote retinal ganglion cells (RGCs) survival and axonal regeneration through the PI3K/Akt system after optic nerve injury in goldfish [16]. These data strongly indicate that IGF-I is an important molecule for controlling regeneration after nerve injury. Furthermore, Maurel and Salzer [17] found that inhibition of PI3K activation completely blocked Schwann cell proliferation and survival. Collectively, we investigated the PI3K/Akt signal pathway mediating IGF-I-induced survival and proliferation in Schwann cell in response to the earthworm extract stimulus.

With a history of several thousand years, the pharmacology and clinical application of traditional Chinese medicine has been well documented. Several Chinese medicines have been identified as enhancing neuron regeneration [18]. Therefore, neuron regrowth induction using Schwann cells and herbal medicine has good potential for treating injured nerves. The Dilong is a widely used Chinese herbal medicine and has been shown to have a dense nutritional content [19, 20]. Previous Dilong studies have shown its antithrombotic [21], hepatoprotective [19], anticancer [22] and scar wound healing characteristics [23]. Lumbrokinase, a novel proteolytic enzyme [24], extracted from the Dilong, has been used to treat stroke and cardiovascular diseases [25]. Moreover, Dilong tissue homogenates have revealed a glycolipoprotein mixture referred to as G-90, composed of macromolecules. The G-90 mixture contains IGFs, immunoglobulin-like growth factor, serine proteases and epidermal growth factor (EGF) [26–31]. Previous studies on G-90 have shown that it promotes cell proliferation [27] and adhesion [29], has antibacterial [32], fibrinolytic and anticoagulant activities [26], and stimulates the cellular immune system [29]. *In vivo*, nanogram quantities of G-90 applied directly to skin wounds have been found to stimulate the synthesis of epidermal growth factor (EGF) and fibroblast growth factor (FGF) and to increase cell proliferation, leading to wound healing [33]. Furthermore, it was also shown that G-90 contains IGF that induced cell proliferation *in vitro* [27]. Recently, experiments further found that a mixed prescription of liquid extracted from Dilong was more effective at improving sciatic nerve function index, motor nerve conductive velocity and the number of myelinated sciatic nerve fibers of regenerated nerve after injury [34]. Whether Dilong has a nerve survival effect and proliferation-enhancing effect on Schwann cell is unknown. This study investigated the mechanisms by which Dilong promotes proliferation and survival of Schwann cell.

## 2. Methods

### 2.1. Dilong Extraction

Spray-dried powder of Dilong, *Pheretima aspergillum* (*Annelida, Oligochaeta, Lumbricidae*), was purchased from Wann Guo Pharmaceutical Co., Ltd, Tainan, Taiwan, Republic of China, Cat. 2467. Dilong powder (2 g) was dissolved in 10 ml of 70% ethanol and left at room temperature for 24 h. The next day the clear supernatant fraction was collected after centrifugation at 370 g (Kubota, Tokyo, Japan) for 20 min. The solvents were then evaporated in a water bath at 37°C for 4 h. The extract was centrifuged for 5 min at 2290 g at 4°C and the supernatant was filtered through a 0.22-*μ*m microspin filter just prior to the experiments. The concentrations used for RSC96 cells treatment in the *in vitro* model were 0, 31.25, 62.5, 125, 250, 500 and 1000 *μ*g ml^−1^. All solutions were stored at −80°C. 

### 2.2. Cell Culture and Treatments

RSC96 cells were purchased from the American Type Culture Collection (ATCC) and cultured in Dulbecco's modified Eagle's medium (DMEM) supplemented with 10% fetal bovine serum (FBS), 4 mM l-glutamate, 1.5 gl^−1^ sodium bicarbonate and 1% non-essential amino acids (NEAA) in a humidified atmosphere of 5% CO_2_ and 95% air. RSC96 cells cultures were treated at the indicated times with indicated concentrations of Dilong extract.

### 2.3. MTT

Cell viability was estimated using a colorimetric assay based on the conversion of tetrazolium dye (MTT) into a blue formazan product. All procedures were described in our previous study [35]. After harvesting and washing twice with phosphate buffered saline (PBS), the cells were cultured in phenol red-free DMEM (1 ml) with MTT (0.5 mg ml^−1^) at 37°C for 4 h. The cells were incubated in isopropenol (1 ml) with shaking for 10 min. Samples were aspirated and measured spectrophotometerically at 570 nm.

### 2.4. Western Blot

Cultured RSC96 cells were scraped and washed once with PBS. The cell suspension was then spun down, and cell pellets were lysed for 30 min in the lysis buffer (50 mM Tris (pH 7.5), 0.5 M NaCl, 1.0 mM EDTA (pH 7.5), 10% glycerol, 1 mM BME, 1% IGEPAL-630 and proteinase inhibitor cocktail (Roche, Mannheim, Germany)) and then centrifuged at 12 000 *g* for 10 min. The supernatants were removed and placed in new Eppendorf tubes for western blot analysis. Proteins from the RSC96 cells were separated in 12% gradient SDS-PAGE and transferred onto nitrocellulose membranes. Nonspecific protein binding was blocked in the blocking buffer at room temperature for 1 h (5% milk, 20 mM Tris-HCl, pH 7.6, 150 mM NaCl and 0.1% Tween 20). The membranes were incubated in 4°C blocking buffer overnight with specific antibodies (1 : 2000) against IGF-I, IGF-IR (Abcam, Cambridge, UK), PI3K and Bcl_2_ (BD Biosciences, San Jose, CA), pAkt (Cell Signaling Technology, Inc., Beverly, MA), pBad, PCNA, cyclin D1, cyclin E, cyclin A and tubulin (Santa Cruz Biotechnology, Inc., Santa Cruz, CA). For repeated blotting, nitrocellulose membranes were stripped with Restore western blot stripping buffer (Pierce Biotechnology, Inc, Rockford, IL, USA) at room temperature for 30 min. Densitometric analysis of immunoblots was performed using the AlphaImager 2200 digital imaging system (Digital Imaging System, CA, USA).

Experiments were performed in triplicate.

### 2.5. Flow Cytometry

Cells were suspended in phosphate buffered saline (PBS, pH 7.4) and fixed with 70% (v/v) ethanol at −20°C for 12 h–16 h. After the ethanol was removed, the cells were washed with PBS and then stained for 30 min with 0.005% propidium iodide (PI). Cellular PI content was measured on a BD FACSCalibur cytometer and data were analyzed using Modfit LT software.

### 2.6. siRNA

Double-stranded siRNA sequences targeting PI3K mRNA were obtained from Dharmacon. A non-specific duplex (Dharmacon, Cat. D-001810-10-20) was used as a control. RSC96 cells were cultured in 60-mm well plates in DMEM without fetal bovine serum. Transfection of PI3K siRNA (Dharmacon, Cat. L-080078-00) was carried out with DharmaFECT Duo transfection reagent (Dharmacon, Inc., Lafayette, CO) according to the manufacture's directions. Specific silencing was confirmed by immunoblotting with cellular extracts after transfection.

### 2.7. Statistical Analysis

Statistical differences were assessed using one-way ANOVA. *P*-value < .05 was considered statistically significant. Data are expressed as the mean ± SD.

## 3. Results

### 3.1. Time-Dependent Proliferation and Survival of RSC Cells Treated with Dilong

**Figure 1:**
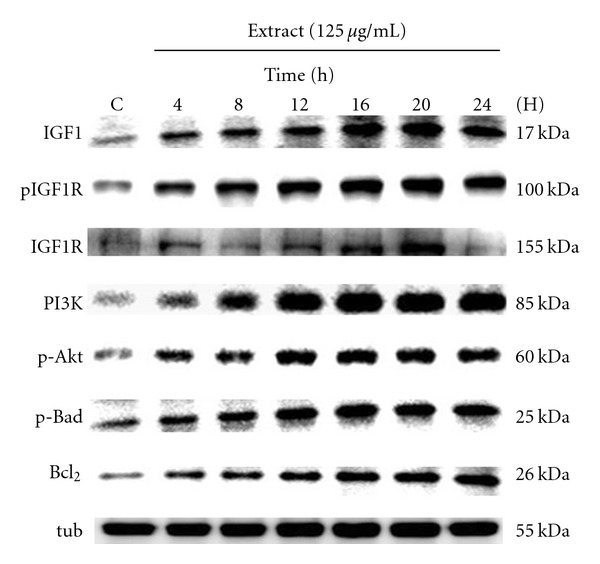
IGF-I-mediated PI3K/Akt signal pathway activation time course for Schwann cell treated with Dilong extract. RSC96 cells were treated with 125 *μ*g ml^−1^ Dilong extract for different times as indicated, and the protein expression of IGF-I was determined by western blot. *α*-tubulin was used as a loading control.

Our previous MTT assay revealed that treatment with 125 *μ*g ml^−1^ Dilong extract for 24 h significantly enhanced RSC96 cell survival and proliferation [36]. Western blot analysis showed that the same treatment strongly promoted IGF-1 protein-mediated cell survival and proliferation (Figure 1). Dilong extract induced IGF-1 activation significantly and rapidly induced the downstream protein expression of the PI3K/Akt system in a time-dependent manner. These results indicate that Dilong may promote Schwann cell survival and proliferation via an IGF-I-mediated signal pathway.

### 3.2. Dilong Treatment Promotes Cell Cycle G_1_ Progression

**Figure 2:**
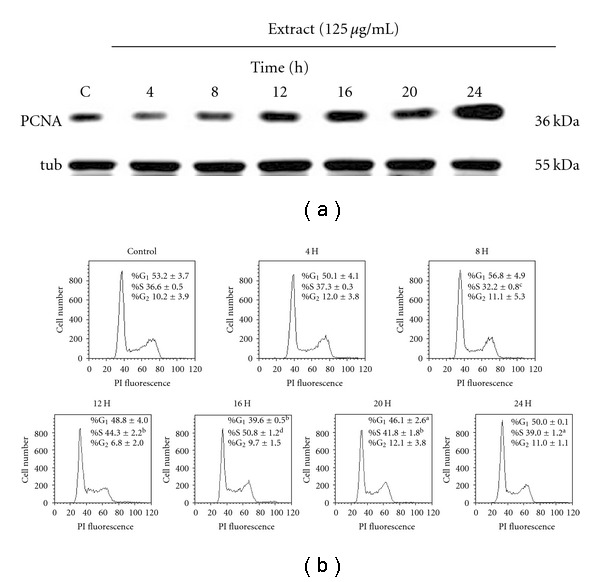
Dilong extract induce the expression of PCNA and promote G_1_ progression. RSC96 cells were stimulated for 24 h with 125 *μ*g ml^−1^ Dilong extract. The protein expression of PCNA was determined by western blot. (a) *α*-tubulin was used as a loading control. Cell cycle distribution was analyzed using flow cytometry. (b) Data (percentage of cells in the indicated phases) are presented as means ± SD of three independent measurements. ^a^ *P *< .05, ^b^ *P *< .01, ^c^ *P *< .001, ^d^ *P *< .0001 versus the control group.

To gain insight into the mechanism by which the extract of Dilong promotes cell proliferation, we used western blot and flow cytometry to determine the proliferating cell nuclear antigen (PCNA) protein levels and cell cycle distribution. The results showed a time-dependent increase in the expression of PCNA (Figure 2(a)). The representative flow histograms depicting cell cycle distribution in Schwann cell cultures following different time exposures to the extract of Dilong are shown in Figure 2(b). Schwann cell exposure to the extract of Dilong led to a significant stimulation of DNA synthesis as evidenced by the increase in the number of S phase cells and decrease in the number of G_1_ phase cells at 12 h–16 h. This finding suggests that Dilong accelerated the G_1_ phase of the cell cycle.

### 3.3. Dilong Induces Expression of Cell Cycle Proliferative Proteins

**Figure 3:**
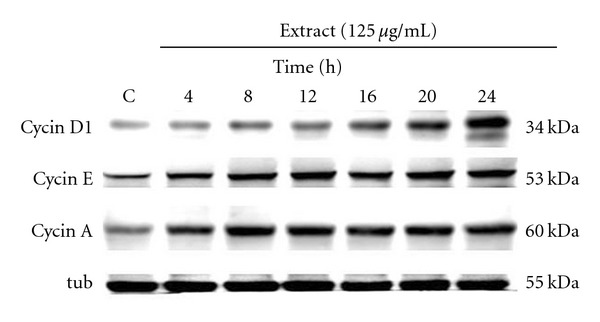
Dilong extract induces the expression of proteins involved in the cell cycle in a time-dependent manner. RSC96 cells were stimulated for 24 h in the presence of 125 *μ*g ml^−1^ Dilong extract. The protein expression of cell cycle regulatory proteins cyclin A, cyclin D1 and cyclin E were determined by western blot. *α*-tubulin was used as a loading control.

Cell cycle progression is tightly regulated by a complex network of cell cycle regulatory molecules, such as cyclins. To elucidate how Dilong modulates the cell cycle to promote Schwann cell proliferation, we used western blot to identify key cell cycle proteins (Figure 3). Consistent with the cell cycle and IGF-I-mediated pathway data presented above, the extract of Dilong treatment induced the upregulated expression of cyclin D1, cyclin E and cyclin A protein in a time-dependent manner. Taken together, these results suggest that Dilong induces Schwann cell proliferation by stimulating the expression of cyclins involved in cell cycle progression.

### 3.4. RSC Cell Survival and Proliferation Enhanced by Dilong Are Mediated through IGF-I and PI3K/Akt Signaling

**Figure 4:**
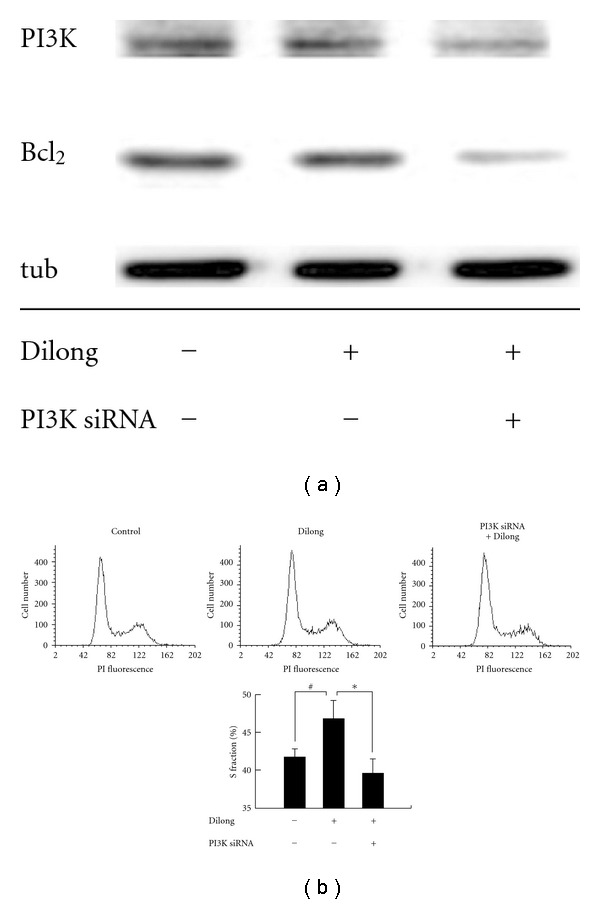
PI3K knockdown inhibited Dilong extract-induced survival and proliferation. Schwann cell was transiently transfected with 100 nM PI3K siRNA for 8 h before Dilong extract treatment. After incubation with 125 *μ*g ml^−1^ Dilong extract for 24 h, cells were harvested and analyzed by immunoblotting using antibodies against anti-PI3K antibody and Bcl_2_. (a) *α*-tubulin was used as a loading control. Cell cycle distribution was analyzed by flow cytometry. (b) Data (percentage of cells in the indicated phases) are the means ± SD of three independent measurements. ^#^compared to control group, *P* = .066; *compared to Dilong-treated group, *P* = .037.

To identify the signaling cascades involved in IGF-I-mediated survival and proliferation of Schwann cells, we transiently transfected cells with PI3K siRNA. Results of the immunoblotting assay showed that PI3K siRNA blocked the extract of Dilong-induced expression of the anti-apoptotic proteins pBad and Bcl_2_ (Figure 4(a)). Knockdown of PI3K led to a significant inhibition of DNA synthesis in cells treated with the Dilong extract for 24 h as evidenced by the fact that the number of S phase proliferating cells decreased (Figure 4(b)). These data indicate that Schwann cell survival and proliferation are PI3K-dependent processes that are mediated, at least in part, by IGF-I.

## 4. Discussion

**Figure 5:**
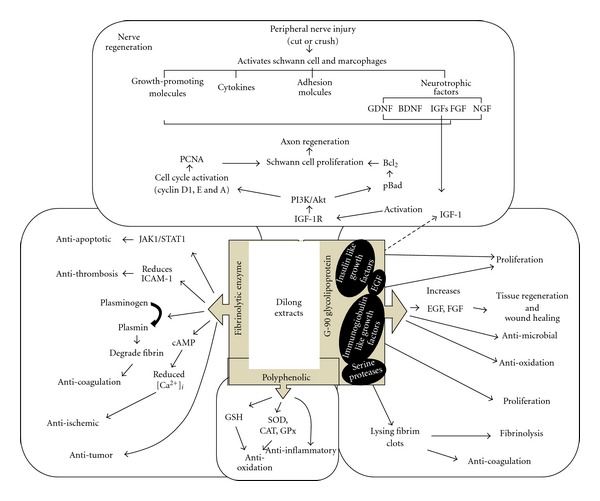
Schematic model of the survival and proliferative effects of Dilong extract on RSC96 Schwann cell. Stimulation of Schwann cell with Dilong extract activate IGF-I signaling, leading to upregulation of the PI3K/Akt pathway and activation of the cell cycle regulatory proteins cyclin D1, E and A, resulting in the survival and proliferation of RSC96 Schwann cell. Dotted lines indicate the hypothetical molecular mechanism of the bioactive compound present in Dilong powder.

This study was undertaken to gain insights into the mechanism by which Dilong extract promotes survival and proliferation of Schwann cells. The coordinated events involved in this mechanism are presented in Figure 5. Schwann cells in the injured nerve area proliferate and form a B*ϋ*ngner band, which supports axonal regrowth [2]. Recent studies have demonstrated that IGF-I plays a crucial role in nerve cell proliferation [10] and survival [16]. However, the molecular mechanism by which Dilong extract induces proliferation and promotes survival of Schwann cells is unknown. This study is the first to demonstrate that extract of Dilong promotes Schwann cell proliferation and survival in a time-dependent manner by stimulating an increase in PCNA (a protein that is expressed by cells during DNA synthesis [37]). Dilong extract-induced Schwann cell proliferation and phosphorylation of the PI3K/Akt signal pathway were both attenuated by transfection of PI3K siRNA. These assays allowed us to examine the individual steps in the complex signaling cascade and clearly illustrate how Dilong affects Schwann cell survival and proliferation.

Chinese herbal medicines have attracted a great deal of attention as alternative and supplemental medicines [38]. Dilong extract has been shown to have different biological properties. It has also been shown to remove stasis (as a slowing or pooling of the blood [39]) by promoting blood circulation [40] and to enhance wound healing [23]. Lumbrokinase is a group of fibrinolytic enzymes isolated from Dilongs [22, 41]. Recent studies have shown that the fibrinolytic enzymes could dissolve blood fibrin clots [42]. Its therapeutic and preventive effects for thrombosis-related disease have been clinically confirmed [43]. The anti-inflammatory activity together with anti-oxidant properties seems to be due to the high polyphenolic content in Dilong tissue [44]. Another critical component of Dilong extract is G-90, a glycolipoprotein mixture that exhibits numerous biological activities. These include mitogenic [27], antibacterial [32], fibrinolytic and anticoagulant activities [26], as well as an antioxidative effect [45]. After treating cell cultures with H_2_O_2_ for 4 h, G-90 allowed the cells to recover and stimulated their growth. Grdisa and Popvic et al. suggest that G-90 can stimulate the growth of fibroblasts and epithelial cells and that it exerts an antioxidant activity, both of which could play a role in its effect on tissue repair. G-90 could be a useful wound-healing agent [45], and it possesses several growth factors including IGF [27]. Recent studies have shown that it is non-allergenic and non-toxic [28], and that it participates in tissue regeneration [30].

The slow rate of peripheral nerve regeneration in humans can lead to prolonged denervation of end organs, raising the specter of permanent paralysis [46]. Neurotrophic factors are a family of growth factors that support and influence the growth and regenerative capacity of neurons [47–50], such as IGFs [51]. Previous studies have demonstrated that the regeneration of mammalian peripheral nerves is dependent on IGFs [52, 53]. Regenerating peripheral nerves express prominent IGF-I in their advancing growth cone. It is obvious that the increased staining of the regenerating nerve is due to IGF-I in the Schwann cell [54], and this increase was associated with the proliferation of Schwann cell [55]. IGF-I, signaling through the type I IGF receptor (IGF-IR), exerts potent effects on neuronal growth and survival [56]. The PI3K/Akt signaling activated by IGF-I through IGF-IR is well known as cell proliferation and survival important pathway [11, 14, 57, 58]. The signal cascade for upregulation of IGF-I is responsible for cell survival of retinal ganglion cells (RGCs) after injury, and it directly induces neurite outgrowth via a PI3K/Akt-dependent mechanism [16, 59].

Moreover, IGF-I rescues Schwann cell from apoptosis via PI3K signaling which is upstream from caspase activation [60], and also requires that PI3K/Akt-mediated progression from G_1_ to S phase of the cell cycle [57]. Conversely, PI3K inhibitors blocked the anti-apoptotic and protective effects of IGF-I, demonstrating that PI3K is essential for trophic factor-induced survival of Schwann cell [58, 60]. We suggested that IGFs from G-90 mixture could promote proliferation and survival by PI3K/Akt pathway activation. These bioactive compounds may directly mediate through the upregulation of IGF-I to induce the phosphorylation of PI3K/Akt pathway and subsequently promotes expression of the anti-apoptotic proteins (pBad and Bcl_2_), PCNA and G_1_ to S phase cell cycle progression, resulting in Schwann cell proliferation and survival promotion in nerve regeneration. Based on these facts, we believe that certain components of Dilong extract have cell proliferation and survival activity. Our results demonstrate that Dilong stimulates Schwann cell proliferation and survival through the PI3K/Akt system mediated by IGF-I.

Proliferating cells pass through several cell cycle checkpoints, such as the G_1_ to S transitions. The former checkpoint is considered to be the most important one in the replication of DNA and mitosis. We thus logically speculate that the extract of Dilong affects Schwann cell proliferation via alterations in the cell cycle progression. Progression through the first gap phase (G_1_) requires cyclin D and cyclin E activity [61]. Expression of these two cyclin proteins orchestrate the progression of cells through G_1_ and into the S-phase of the cell cycle [62, 63]. Cyclin A is required for DNA replication in the S-phase and in mitosis initiation (M-phase) [63]. Our data show that Dilong extract induces cell cycle progression at the G_1_ to S phase transition. The time course study revealed that Dilong extract promoted DNA replication and growth of RSC96 cells by upregulating the sequential expression of cyclin D1, cyclin E and cyclin A, thereby elevating the number of cells in the S phase in a time-dependent manner. Studies on the proliferative action of IGF-I in cultured fibroblasts [64, 65] and mammary epithelial cells [66] indicate that IGF-I acts to stimulate progression through G_1_ or the G_0_/G_1_ transition. Our data are in agreement with the results of those studies that the cell cycle is not only regulated by cyclins but is also mediated by IGF-I. Collectively, we suggest that cell cycle alterations may be critical determinants of the increased proliferation potency of Dilong extract.

The IGF-IGFIR-Akt-Bcl_2_ axis stimulates tissue growth [67] and axonal regeneration [16]. IGF in certain cells, such as hematopoietic cells, functions as an inhibitor of cell death [68]. We suggest that the extract of Dilong promotes proliferation by allowing Schwann cell survival. Activation of the PI3K/Akt pathway promotes cell survival. Activation of Akt leads to the phosphorylation of Bad [69] and connects a proximal survival signal with the Bcl-2 family to protect against apoptosis. Although we cannot rule out the possibility that the other minor candidates in the pathways, we unequivocally showed that PI3K/Akt signal proteins were upregulated at least in part by IGF-I in RSC96 cells treated with Dilong extract.

In conclusion, the findings of our study provide a potential mechanism by which Dilong extract promotes neuron regeneration. We found that cell proliferation and survival of Schwann cells are mediated by the upregulated expression of IGF-I and activation of the PI3K/Akt signal pathway. The activity of Dilong extract is probably related to its ability to induce G_1_ phase cell cycle progression by altering the expression of proteins that control the cell cycle, resulting in the upregulation of the anti-apoptotic protein. Further analyses are needed to determine the presence of other bioactive compounds in Dilong extract that might promote cell survival and proliferation.
